# 7 Tesla MRI Reveals Brain Structural Abnormalities and Neural Plasticity in RPGR-Related Retinitis Pigmentosa

**DOI:** 10.3390/jcm14051617

**Published:** 2025-02-27

**Authors:** Katarzyna Nowomiejska, Katarzyna Baltaziak, Aleksandra Czarnek-Chudzik, Michał Toborek, Anna Niedziałek, Katarzyna Wiśniewska, Mateusz Midura, Robert Rejdak, Radosław Pietura

**Affiliations:** 1 Department of General and Pediatric Ophthalmology, Medical University of Lublin, 20-079 Lublin, Poland; k.baltaziak@gmail.com (K.B.); olaczarnek@poczta.fm (A.C.-C.); robertrejdak@yahoo.com (R.R.); 2 Radiography Department, Medical University of Lublin, 20-093 Lublin, Poland; toborekmichal@gmail.com (M.T.); anianiedzialek@interia.pl (A.N.); katarzyna.agnieszka.wisniewska@gmail.com (K.W.); radoslaw.pietura@umlub.pl (R.P.); 3 Faculty of Electronics and Information Technology, Institute of Radioelectronics and Multimedia Technology, Warsaw University of Technology, 00-661 Warszawa, Poland; mateusz.midura.dokt@pw.edu.pl

**Keywords:** 7 Tesla MRI, RPGR-related RP, brain plasticity

## Abstract

**Objectives:** The purpose was to quantitatively examine brain structures using 7 Tesla MRI in the presence of visual loss caused by retinitis pigmentosa (RP) related to retinitis pigmentosa GTPase regulator (RPGR) gene pathogenic variants. **Methods:** Twelve male patients with RP (mean visual acuity 0.4) related to confirmed RPGR pathogenic variants and fifteen healthy volunteers were examined with 7 Tesla MRI of the brain. Measures of the lateral geniculate nucleus (LGN) volume were performed manually by three independent investigators (radiologists) using ITK-SNAP (Insight Segmentation and Registration Toolkit) software. Other brain structures were evaluated using the open-source automated software package FreeSurfer. Prior to the 7 Tesla MRI, patients underwent an ophthalmic examination and a 1.5 Tesla MRI. **Results:** The mean LGN volume (right—100 mm^3^, left—96 mm^3^) and left lingual gyrus volume (6162 mm^3^) were significantly lower in RPGR patients in comparison to the control group (129 mm^3^, 125 mm^3^, and 7310 mm^3^, respectively), whilst some brain regions related to other sensory information such as the left isthmus cingulate (3690 mm^3^) and entorhinal cortex (right—1564 mm^3^, left 1734 mm^3^) were significantly or almost significantly higher in the RPGR group than in the control group (2682 mm^3^, 960 mm^3^, and 1030 mm^3^, respectively). Moreover, compared to the control group, the RPGR group’s thalamus-to-LGN ratio was substantially higher. **Conclusions:** The use of the 7 Tesla MRI revealed numerous structural abnormalities of the visual pathway in patients with RPGR-related RP. The reorganization of the structures of the brain demonstrated in patients with RPGR-related RP reveals a certain degree of plasticity in response to visual loss. These findings may help improve diagnostic and therapeutic strategies for RP patients and contribute to the development of precision medicine.

## 1. Introduction

The RPGR (retinitis pigmentosa GTPase regulator) gene (OMIM 312610) accounts for the most frequent genetic type of X-linked retinitis pigmentosa (XLRP) [1]. X-linked forms of RP account for 10 to 20% of all RP cases [2] and primarily affect the rod photoreceptors [3]. Pathogenic variants in the RPGR gene are the most common causes of XLRP, responsible for over 70% of XLRP [4]. In the first RPGR mutation screening in Polish patients, the prevalence of RPGR pathogenic and likely pathogenic variants in the studied cohort of male patients with an RP phenotype was 18% [5]. The disease manifests severely in males with RPGR mutations, with an early onset of night blindness and a progressive constriction of the visual field, often causing patients to become legally blind by the age of 30 to 40 years. Pathogenic variants in the RPGR gene result in the progressive degeneration of rod and cone photoreceptors. Consequently, the remodeling of the neural retina occurs, including bipolar and horizontal cell deafferentation and a retraction of their dendrites [6]. In spite of retinal remodeling, mechanisms resembling developmental and central nervous system plasticity occur [6].

Currently, there is no effective treatment for RP; however, there are efforts focused on research in novel therapeutic strategies, including pharmacological targeting, gene augmentation therapy, cell transplants, and electronic prostheses [7,8]. All of these strategies depend on the functional integrity of the visual system downstream of photoreceptors. Clinical trials in this area require objective and quantitative assessment methods in order to evaluate the visual function, which are difficult to perform in advanced visual field loss due to RP.

The visual pathway begins with the photoreceptors in the retina; light enters the eye and is converted into neural signals. These signals are then sent by the retinal ganglion cells along the optic nerve to the lateral geniculate nucleus (LGN). From the LGN, signals are propagated along the optic radiation to the primary visual cortex (V1). From V1, the central visual signal path begins to go quickly to the areas of the brain that specialize in visual function.

The visual pathway of patients with retinal dystrophies, including RP, has not been examined with 7 Tesla MRI so far. Over the last ten years, various brain imaging techniques have been used, such as manual segmentation, diffusion tension imaging, and surface- and voxel-based morphometry, to demonstrate neural plasticity in the “visual cortex” of blind people [9].

Visual prostheses are promising devices for RP patients and serve to restore visual function caused by acquired blindness, and these arrays can be placed in any part of the early visual pathway, such as the retina, optic nerve, LGN, or visual cortex. The LGN has become an especially attractive target to researchers given that advances in deep brain stimulation have created easy surgical access to the thalamus [10,11]. The compact structure of the LGN supports a wide prosthetic visual field, and the over-representation of the fovea allows for higher acuity vision than other approaches. Current LGN research has proven that stimulation can produce phosphenes and that such stimulation produces similar responses in the visual cortex as natural visual stimulation [12].

Magnetic resonance imaging (MRI) allows noninvasive and variable assessments of the whole visual system from the eye to the visual cortex [13]. However, the structural 1.5 T MRI lacked the resolution and accuracy needed for exact LGN visualization and volume quantification. There are studies showing good preservation of the visual pathways from the retina to the visual cortex in RP [14].

The aim of this study was to investigate quantitatively potential changes in the visual pathway in patients suffering from RP related to RPGR gene pathogenic variants, with an emphasis on LGN.

## 2. Materials and Methods

### 2.1. Patients

This study was a prospective non-interventional one-center case–control study. Approval of the Ethics Committee of the Medical University of Lublin, Poland, was obtained (KE-0254/26/2020). Written informed consent was obtained from all subjects. All methods were performed in accordance with the relevant guidelines and regulations. The inclusion criteria were as follows: a diagnosis of XLRP with confirmed RPGR pathogenic variants and an age of more than 15 years. All patients were confirmed with pathogenic variants of the RPGR gene (Table 1).

Exclusion criteria were as follows: intraocular pathologies that would affect visual acuity or visual field, a history of ocular trauma, or surgery. All patients underwent standard ophthalmic examinations including best-corrected visual acuity using Snellen charts, kinetic perimetry (Octopus 900, Haag-Streit, Koeniz near Bern, Switzerland), slit-lamp examination, wide-field photography (Optos), optical coherence tomography (SD-OCT), and fundoscopy. The mean visual acuity of RP patients was 0.4 (SD ± 0.2), the mean central retinal thickness was 185.25 µm (28.253 µm), and the mean V4e isopter area was 4556.60 deg2 (Table 1). All RPGR patients underwent MRI 1.5 T to exclude other CNS pathologies.

Twelve male patients (mean age 30.2 years; range 15–57 years) with RP related to pathogenic RPGR variants were examined with 7 Tesla MRI of the brain. Additionally, 14 healthy male subjects (mean age 26.1 years; range 20–33 years) were examined. Inclusion criteria for the control group were a best-corrected visual acuity of 20/20 and no history of previous ophthalmologic or neurologic disease.

The study group consisted of 12 individuals (all male), while the control group comprised 14 individuals (3 females, 11 males). The age distribution in both groups was normal (Shapiro–Wilk test; W_S_ = 0.92; p_S_ = 0.25; W_C_ = 0.89; p_C_ = 0.09). Due to the lack of homogeneity of variance (Levene’s test, *p* < 0.001), the Cochran–Cox test was conducted to determine whether age differentiates the groups. The Cochran–Cox test result, t = 1.01 (13.414), was not significant (*p* = 0.33). Therefore, age is not a differentiating factor between the groups and does not affect the outcome variable.

Imaging data were obtained at the ECO-TECH COMPLEX scientific center (Lublin, Poland) using a Discovery MR950 7 T MRI system (GE Healthcare, Chicago, IL, USA) with a gradient strength of 50 mT/m and a slew rate of 200 T/m/s. The coil configuration used for performed examinations was a two-channel birdcage coil driven in quadrature for transmission and a 32-channel array coil for reception (Nova Head 32-channel head coil, 2Tx/32Rx, Nova Medical Inc., Wilmington, MA, USA). The imaging protocol for this study in the 7 T examination contained two sequences: 3D BRAVO T1-weighted and 3D MT-weighted SILENT, obtained with parameters from the table below (Table 2).

**Figure 1:**
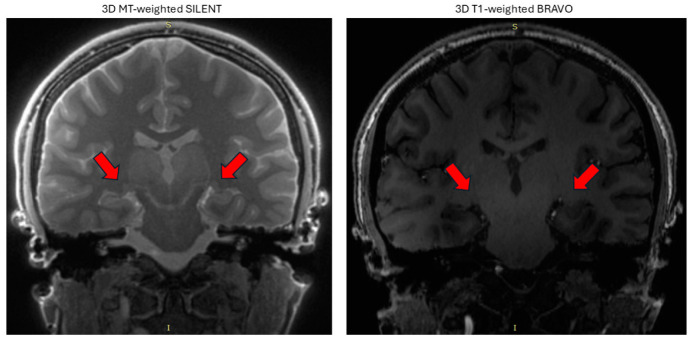
Comparison of lateral geniculate nucleus (red arrows) visibility in two sequences. On the left side is 3D MT-weighted SILENT; on the right side is 3D T1-weighted BRAVO. Images acquired at the Ecotech Complex (Lublin, Poland).

The 3D MT-W SILENT sequence was almost completely free of acoustic noise, had excellent fat suppression, and the scan time was very short. The layer thickness of 1 mm in 3D BRAVO T1-W was equivalent to the layer thickness of 0.8 mm in 3D MT-W SILENT. The visibility of small structures as LGN was much better with 3D MT-W SILENT (Figure 1).

### 2.2. MRI Data Analysis

Volume and cortical thickness analyses were performed using the open-source automated software package FreeSurfer (version 7.4.1, Massachusetts General Hospital, Harvard Medical School; http://surfer.nmr.mgh.harvard.edu, accessed on 20 June 2023). Recon-all stands for reconstruction, as in reconstructing a two-dimensional cortical surface from a three-dimensional volume, which uses a high-resolution image T1-weighted anatomical scan with high contrast between white matter and grey matter. We utilized a version of the recon-all procedure, which incorporates typical data reconstruction steps such as skull stripping, volumetric registration, normalization, volumetric labeling, segmentation, smoothing, and cortical parcellation (https://surfer.nmr.mgh.harvard.edu/fswiki/recon-all, accessed on 20 June 2023). Our voxel size was 1 mm^3^. Finally, tabulated data summarizing the segmented results were gathered using a selection of FreeSurfer summarizing scripts. Volume assessment was performed according to data obtained from the Desikan–Killiany parcellation atlas. The LGN volume was calculated using ITK-SNAP version 4.0.0-rc.2. This software allows the manual delineation of anatomical regions of interest on 3D medical images. A 3D MT-weighted SILENT sequence was evaluated independently by three radiologists (R.P., K.W., M.M.s). Their measurement results were comparable given that they used the same linear image contrast adjustment: minimum 250, maximum 1750, level 1000, window 1500.

### 2.3. Statistical Analysis

All statistical analyses were performed in the Statistica TIBCO 13.3 software. A significance level of *p* = 0.05 was used in each analysis. To determine whether there were differences between the patient and control groups, the analysis of brain regions and LGN was based on parametric tests, i.e., Student’s *t*-test for independent variables, the Cohran–Cox test with separate variance estimation, and the non-parametric Mann–Whitney U-test. First, the Shapiro–Wilk test was performed for each brain parameter (in groups) to decide when to use the parametric *t*-test and when to use the non-parametric Mann–Whitney U-test. If the distributions of the parameter in both groups were normal, the homogeneity of variances was assessed using Levene’s test, and according to the criterion of homogeneity, either the *t*-test or the Cohran–Cox test was selected. LGN was measured three times in each patient. For further analysis, an average of the three measurements was calculated and then reviewed for both lh_LGN and rh_LGN. Student’s *t*-test for independent groups was performed to show differences in LGN volume between groups. A Cohran–Cox test was performed to show LGN differences between groups, as there are no homogeneous variances (Levene’s test, F = 4.44 (*p* = 0.02)).

## 3. Results

**Figure 2:**
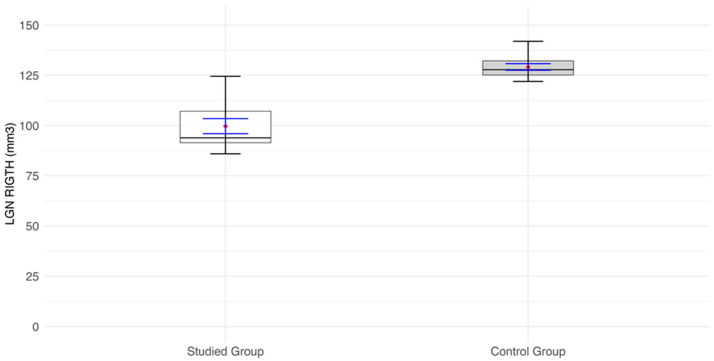
Values of the lateral geniculate nucleus (LGN) volume (mm^3^) of the right side of the visual pathway. Red diamonds indicate the mean values, while blue lines represent confidence intervals.

**Figure 3:**
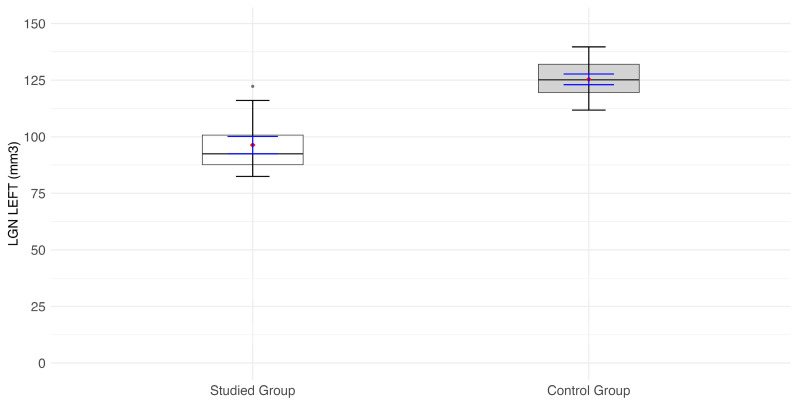
Values of the lateral geniculate nucleus (LGN) volume (mm^3^) on the left side of the visual pathway (rh_LGN). Values of the lateral geniculate nucleus (LGN) volume (mm^3^) of the left side of the visual pathway. Red diamonds indicate the mean values, while blue lines represent confidence intervals.

**Figure 4:**
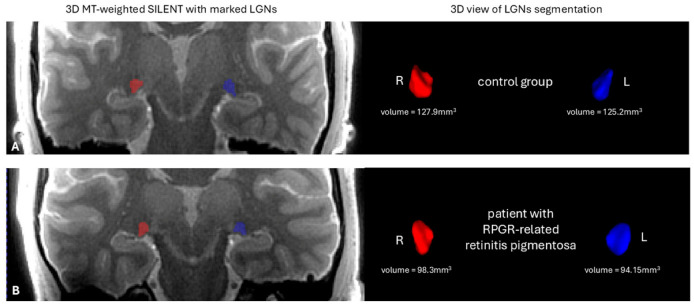
On the left side is the anatomical image of the brain 3D MT-weighted SILENT with marked lateral geniculate nucleus (LGN); on the right side are the 0 3D view results of LGN manual segmentation using ITK-SNAP software. Images show one case chosen from the control group (**A**) and a patient with RPGR-related retinitis pigmentosa (**B**). Images acquired at the Ecotech Complex (Lublin, Poland).

Volumes of LGN of the right and left side (mm^3^) are significantly lower in the RPGR-related RP patient group than in the control group (*p* < 0.0001) (Figure 2, Figure 3 and Figure 4).

In addition to LGN volumes, thalamus volumes (mm^3^) were also calculated. However, there is no correlation between these characteristics (in the control group either). The ratio of the thalamus to LGN was assessed as follows:

w_(2_lh ) = (lh-thalamus)/(lh-LGN * lh_LGN)

w_(2_rh ) = (rh-thalamus)/(rh-LGN * rh_LGN)

The significant result of the Cohran–Cox test, t = 4.06 (*p* = 0.001), indicates that the values of this ratio in the patient group are significantly higher than in the control group. The substantial result of the Cohran–Cox test, t = 3.50 (*p* = 0.0034), shows that the values of this ratio in the patient group are significantly higher than in the control group.

In the patient group, mean right thalamus volumes are 0.72 squared of mean right LGN volumes, while in the control group, mean right thalamus volumes are 0.46 squared of mean right LGN volumes. In the patient group, mean left thalamus volumes are 0.87 squared of mean lh_LGN volumes, while in the control group, mean left thalamus volumes are 0.55 squared of mean lh_LGN volumes.

**Figure 5:**
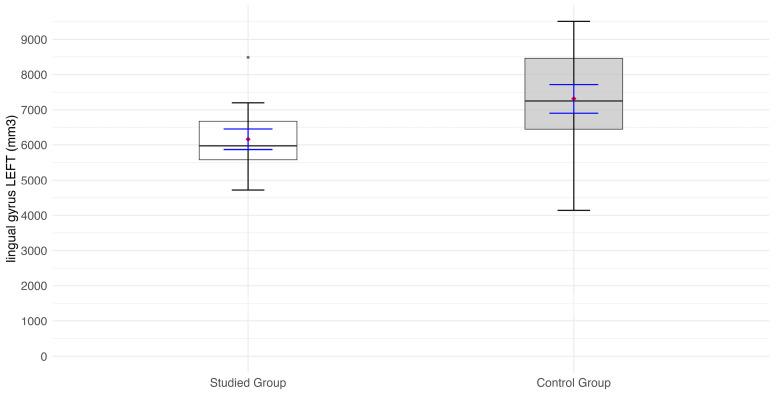
Values of the lingual gyrus volume (mm^3^) on the left side of the visual pathway (lh_lingual). Red diamonds indicate the mean values, while blue lines represent confidence intervals.

The mean values of lingual gyrus volume (mm^3^) are significantly lower both for the right (*p* = 0.002) and left (*p* = 0.037) sides in the patient group than in the control group (Figure 5).

**Figure 6:**
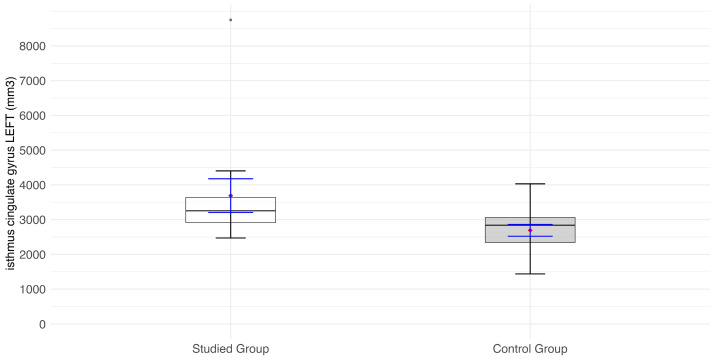
Values of the isthmus cingulate volume (mm^3^) of the left side of the visual pathway. Red diamonds indicate the mean values, while blue lines represent confidence intervals. Statistical significance of obtained results is included in Table 3.

The average values of the left isthmus cingulate gyrus volume (mm^3^) in the patient group are significantly higher than in the control group (*p* = 0.009) (Figure 6).

The differences that involved the following parameters were close to statistical significance: the mean values of right (1564 mm^3^, *p* = 0.051) and left (1737 mm^3^, *p* = 0.049860) volume of entorhinal cortex are higher almost significantly in the patient group than in the control group (960 mm^3^, and 1030 mm^3^, respectively). There were no profound differences in the remaining structures of the brain and the control group.

## 4. Discussion

To the best of our knowledge, this is the first structural study to fully explore visual pathway structures in patients suffering from intermediate RP. We examined the size and properties of the visual system between the retina and the primary visual cortex. The main finding of this study is that the volume of both LGNs and lingual gyrus is decreased in patients suffering from RP related to RPGR gene pathogenic variants. On the contrary, the volume of the left isthmus cingulate is increased in RP patients, in comparison to the healthy control male subjects.

The LGN is a small deep brain structure, a nucleus within the thalamus, typically of a 4–5 mm diameter. LGN receives information from the retinal ganglion cells and projects axons via optic radiations to the primary visual cortex [15]. It has already been established that LGN as determined by 1.5 T MRI decreases with age [16]. Conventional two-dimensional multi-slice imaging, which often uses slice thicknesses of 5 mm or a little less, is poorly suited for providing data for an accurate measurement of such small structures. Three-dimensional imaging, with isotropic resolution, is preferred. The superior Signal-to-Noise Ratio (SNR) of 7T MR permits the acquisition of three-dimensional data with a higher resolution; in this study, we achieved < 0.8 mm. The LGN is typically delineated by at least 5 pixels in each direction, allowing volume measurements with strong resistance to the partial volume effects encountered when segmenting small structures with two-dimensional scans. In previous papers, LGNs have been shown using functional magnetic resonance imaging (fMRI) because MRI 1.5T and 3T had an insufficient resolution to image morphology of small structures like LGNs [17]. MR 7T was used to determine the structural and volumetric anatomy of the LGN. As the first sequence, PDw (proton density-weighted) was used. Then, MP2RAGE was the better sequence used in MR7T for the precise visualization of LGNs morphology, but its small size and deep localization make imaging challenging [18,19,20]. MP2RAGE was a step forward; however, the lack of sufficient contrast in volume quantification was the main limit for the clinical and neuroscientific applicability of LGN imaging [21]. The ratio of the thalamus to LGN was intended to objectify the change in the size of the LGN regardless of the patient’s size (height and weight). The reduction in the volume of the LGN in the study group was assessed absolutely in mm^3^ and relatively as a ratio of the LGN to the thalamus, which is a well-defined and unchanged brain structure in the RPGR patients group and in the control group, and its size depends on the patient’s size (height and weight).

LGN volume has already been measured by 7 Tesla MRI in patients with Leber hereditary optic neuropathy (LHON), demonstrating the effects of anterograde degeneration on LGN volume [22]. LGN has also been found to be reduced in patients with glaucoma [23]. Moreover, the authors concluded that patients with more advanced glaucoma tended to have lower volumes of LGN. LGN has been already found to be correlated with the corresponding ganglion cell layer loss in acquired human post-geniculate lesions [24]. Atrophy of the LGN can be the result of direct damage or trans-synaptic degeneration. The latter can be caused either by anterograde (Wallerian) degeneration from the optic nerve axons [25] or from retrograde neuronal degeneration (TRND) of neurons downstream from the LGN in optic radiations or the primary visual cortex (V1) [26].

The human brain has a natural ability, called neuroplasticity, to adapt itself in response to every perturbation in the external and internal environmental factors. The absence of vision from birth, called visual deprivation, causes massive structural changes that take place not only in the visually deprived cortex but also in other brain areas [27]. Shimony and colleagues found that patients with early blindness had notable atrophied visual radiation, while Yu and colleagues found that the integrity of the white matter of the corticospinal tract was enhanced [21,28]. Park and coworkers [29] observed that the surface range of primary and related visual areas decreased significantly in patients with congenital blindness. Significantly decreased fractional anisotropy values in the group of patients with monocular blindness were shown in the corpus callosum, the right retrolenticular part of the internal capsule, the anterior corona radiation, the superior corona radiation, the posterior corona radiation, the posterior thalamic radiation, the right sagittal stratum (right), and the superior longitudinal fasciculus 1 in 3T MRI.

Anatomic studies have shown a good preservation of the visual pathways from the retina to the visual cortex in patients suffering from RP [30], but much less is known about the visual cortex in those patients. We have demonstrated decreased volume of the lingual gyrus in RP patients with intermediate visual acuity. The lingual gyrus is a part of the medial occipital lobe and is necessary for basic and higher-level visual processing [31]. The lingual gyrus is a cortical structure within the ventral visual stream [9] that is involved in fundamental visual processes such as eye movements, visual sensation, visual cognition [32], and early stages of face processing [31]. It has also been shown that the lingual gyrus plays an important role in color perception [33]. Thus, a decreased volume of the lingual gyrus in sighted RP patients may indicate impaired visual cognition, face processing, and color perception.

Interestingly, RP patients examined in this study displayed a larger volume and surface area of the left isthmus cingulate, similar to schizophrenia patients comorbid with depression [34]. The cingulate cortex is a fundamental part of the limbic system associated with depression, whereas the isthmus cingulate cortex refers to the narrowing of the cingulate cortex connecting the posterior cingulate cortex to the parahippocampal gyrus. Structural imaging studies showed the role of the isthmus cingulate cortex in both major and subthreshold depression, but the function of this structure is not yet well understood [32,35]. It has already been proven that enlargement in the left isthmus cingulate is associated with depressive symptoms in schizophrenia patients [32]. Isthmus cingulate may mediate the interaction between emotion- and memory-related processes, as functional imaging studies have found that emotional stimuli have activated the posterior cingulate cortex, which may suggest that [36]. We can anticipate that the enlargement of the left isthmus cingulate is connected to mood disorders in RP patients. It is already known that vision impairment is psychologically and socially debilitating [37,38]. Moreover, our study showed the differences in the volume of the right and left entorhinal cortices were almost significantly higher than in the control group. The entorhinal cortex is a brain area that serves as the main connection to the hippocampus, playing a crucial role in memory, navigation, and time perception. Axons originating from the entorhinal cortex project to cortical regions surrounding it, reaching even the primary visual cortex [39]. The essential roles of the cingulate and entorhinal cortices in consolidated memory have been shown [40], so the enlargement of these areas of the cortex in RP patients may be explained by the heightened need for long-lasting memory of people with decreased visual acuity.

Many studies have emphasized neuroplastic changes in occipital brain areas [41,42], while other reports have described anatomical changes in other parts of the blind brain, including cortical and subcortical structures such as the LGN, the hippocampus, and the corpus callosum [42,43].

It has been shown that not only is the visual cortex activated by tactile stimuli but the tactile motion and shape information are funneled into the visual pathways [44,45], respectively, with blindness resulting in brain volumetric differences that depend upon the duration of blindness. In a big meta-analysis regarding brain structural changes in early- (EB) and late-onset blindness (LB) of different origins, the results of many studies revealed atrophic changes throughout the whole extent of the retino-geniculo-striate system in both EB and LB, whereas changes in areas beyond the occipital lobe occurred in EB only [9]. According to certain studies, adult subjects with retinal dystrophies still have some plasticity, and they can learn to “see” using artificial visual input [46]. It has already been demonstrated that the adult visual brain possesses some degree of plasticity and is able to reorganize its response after many years of deprivation in adulthood [46]. We can speculate that the other senses are trying to compensate for the loss of vision and that this process is associated with a reorganization of the brain through cross-modal plasticity [47]. It is already known that the visual system occupies about one-third of the surface of the cortical mantle, so other sensory modalities will be recruited to accommodate [48].

This study has some limitations, especially in regard to inclusion criteria. First, only male patients with intermediate visual acuity due to RP were included and this selection might affect the generalizability of the findings. Only patients with X-linked RP and RPGR gene pathogenic variants were examined; thus, the investigated group was quite homogenous. Patients included in the present study were not completely blind, only two patients had visual acuity lower than 0.1, and they were quite young (mean age of 30 years). Blindness can occur later in life in these patients, as RP is a progressive disease. Thus, the visual system of these patients has developed normally until vision loss, and they basically possess a visual brain quite similar to that of seeing people. The neuroplastic processes of the brain that accompany the onset of blindness are less strong in late blindness, regarding the fact that plasticity is highly dependent on critical periods of development [48]. The reorganization of the blind brain demonstrates the multisensory nature of brain plasticity that can arise when development is irregular [28,49]. As the investigated group in this study is quite limited, we are performing further investigations in a larger group of patients suffering from RP with another genotype and with a wide range of visual acuity, gender, and age.

Recent progress has provided many promising perspectives, including the development of gene therapy for IRD. Patients with RPE65-associated dystrophies took advantage of gene replacement and augmentation therapy using subretinal injections of adeno-associated virus (AAV) vectors with genetically incorporated RPE65 cDNA. Currently, several human clinical trials are underway and are at various stages of completion. A wide range of approaches towards sight restoration are currently being pursued, including cortical, LGN, and retinal prostheses [50,51]; optogenetic techniques [52]; and gene therapy [53]. A better understanding of the modulation of neuroplasticity is essential for the development of rehabilitation schemes and technologies for low-vision patients who undergo new therapies. We believe that a quantitative volumetric assessment of the LGN and other visual pathway structures will allow for further research to better understand the dynamics of RP along with other retinal dystrophies, including relations to new rehabilitation and treatment methods. Further studies are needed, particularly those concerning blind patients suffering from RP with different genotypes. Moreover, future research should aim to include a more diverse patient population to better evaluate the prevalence of brain structural abnormalities and their relationship with RP.

## Figures and Tables

**Table 1:** Patient ages (all patients were males) as well as pathogenic variants and visual acuity (Snellen decimal notation).

Patient ID	Age (Years)	Variant in the RPGR Gene	Visual Acuity— Right Eye	Visual Acuity— Left Eye
1.	29	c.2442_2445del; p.Gly817fs—pathogenic in ORF15	0.7	0.7
2.	39	c.2340_2341del; p.Arg780fs—pathogenic in ORF15	0.01	0.01
3.	34	p.Glu 863 -likely pathogenic in ORF15	0.5	0.6
4.	20	c.2389dup p.Glu797fs; —likely pathogenic in ORF15	0.7	0.7
5.	20	p.Glu797fs; c.2389dup—likely pathogenic in ORF15	0.4	0.2
6.	23	c.2455dup p.Val819fs—likely pathogenic in ORF15	0.6	0.3
7.	15	c.2323_2324delAG; p.Arg775Glufs 59—pathogenic	0.2	0.5
8.	17	c.2270_2343dup; p.Gly782Argfs 58—likely pathogenic		
9.	35	c.2966delA; p.Glu989Glyfs 100 likely pathogenic	0.2	0.4
10.	20	c1905 + 413del; p.Gly773GlufsTer42—pathogenic in ORF15	0.3	0.4
11.	56	c.1245 + 1G > A; p-likely pathogenic	0.6	0.5
12.	44	c.2236_2237del; p.Glu746ArgfsTer23—pathogenic	0.05	0.05

**Table 2:** Imaging protocols used in the present study for 7 Tesla MRI of the brain. Abbreviations: FOV (field of view), TE (echo time), TR (repetition time), TI (inversion time), and NEX (number of excitations).

	3D BRAVO T1-W	3D MT-W SILENT
FOV [cm]	22 × 22	17.6 × 17.6
Slice thickness [mm]	1.0	0.8
TE [ms]	2.6	0.0
TR [ms]	6.6	257
TI [ms]	450	not applicable
Matrix size	288 × 288	224 × 224
NEX	1	3
Flip Angle	12	2

**Table 3:** Statistical significance of obtained results (LGN—lateral geniculate nucleus, S—studied group, C—control group, SD—standard deviation).

	Group	Mean (mm^3^)	SD (mm^3^)	Median (mm^3^)	Levene’s Test		*t* Test		95 Percent Confident Interval	
					F	p	t (df)	*p* Value	Left	Right
**LGN left**	**S**	96.3	12.79	92.5	0.89	0.90	−6.66; (22)	<0.001	−38.08	−20.00
	C	125.4	8.43	125.5						
**LGN right**	S	99.7	12.46	93.9	2.73	0.11	−7.61; (22)	<0.002	99.69	129.18
	C	129.2	5.91	127.8						
**Lingual gyrus left**	S	6162.4	1021.30	5971.5	0.86	0.36	−2.28 (22.76)	0.03	−2190.99	−104.31
	C	7310.10	1529.79	7245.5						
**Isthmus cingulate gyrus left**	S	3690.50	1683.87	3255.00	1.98	0.17	−7.61; (22)	<0.002	99.69	129.18
	C	2681.90	637.03	2837.50						

## Data Availability

The datasets analyzed during the current study may be made available from the corresponding author upon reasonable request.
